# Reliability and quality of educational content on sleep apnea-hypopnea syndrome: A cross-sectional content analysis on TikTok and Bilibili

**DOI:** 10.1097/MD.0000000000049438

**Published:** 2026-06-26

**Authors:** Shan Wang, Ying Mao, Fang Wang, Jiaqi Li, Zhenxing Zhang

**Affiliations:** a Department of Gastrointestinal Surgery, Shaoxing People’s Hospital, Shaoxing, China; b Department of Special Examination, Shaoxing People’s Hospital, Shaoxing, China

**Keywords:** Bilibili, health information quality, short-video platforms, sleep apnea-hypopnea syndrome, TikTok

## Abstract

Sleep apnea-hypopnea syndrome (SAHS), a prevalent sleep-disordered breathing disease, burdens global health. In the digital era, short-video platforms such as TikTok and Bilibili have become a major source of health information for the public, but their quality is scarcely studied, raising accuracy and reliability concerns. This study aimed to systematically evaluate the reliability and quality of SAHS educational videos on TikTok and Bilibili using validated tools, such as modified DISCERN (mDISCERN), Global Quality Score (GQS), and Journal of the American Medical Association benchmark criteria (JAMA), and to analyze the associations between content quality, uploader types, and user engagement metrics. A cross-sectional analysis was conducted by retrieving the top 150 videos from each platform using “Sleep Apnea-Hypopnea Syndrome” as the keyword. After excluding duplicates and irrelevant videos, 274 videos (150 from TikTok, 124 from Bilibili) were analyzed using the GQS, the mDISCERN, and the JAMA. Video characteristics, uploader identity, content coverage, and user engagement metrics were evaluated and compared across platforms and uploader types. TikTok videos were significantly shorter but received higher user engagement (likes, comments, shares, and collections) compared with Bilibili videos. Healthcare professionals were the primary uploaders on TikTok (57%), whereas individual users dominated on Bilibili (54%). Video quality scores (GQS, mDISCERN, JAMA) were significantly higher on TikTok than on Bilibili (*P* < .001). Videos uploaded by healthcare professionals scored the highest in quality and reliability. Strong positive correlations were found among the engagement metrics, but only weak correlations existed between engagement and quality scores. TikTok had higher engagement and better quality than Bilibili, but the overall video quality of the SAHS content on both platforms still needs improvement. Healthcare professional-uploaded videos are more reliable. These findings highlight the need for better regulation and monitoring of health content on short-video platforms.

## 1. Introduction

Sleep apnea-hypopnea syndrome (SAHS) is a common sleep-related respiratory disorder characterized by recurrent partial or complete upper airway obstruction during sleep, leading to decreased blood oxygen saturation, fragmented sleep, and excessive daytime sleepiness.^[1,2]^ SAHS can be divided into 3 types: obstructive, central, and mixed. In clinical practice, obstructive SAHS is one of the most common forms of SAHS, accounting for approximately 80% of SAHS cases.^[3]^ With the prevalence of obesity and aging of the population, the number of patients with SAHS is increasing year by year.^[4]^ According to the available epidemiological data, 5.7% to 6.0% of adult males and 2.4% to 4.0% of adult females suffer from SAHS.^[5,6]^ In addition to directly affecting sleep quality, SAHS is also closely associated with a range of serious health complications, including systemic hypertension, cardiovascular disease, metabolic disorders, neurocognitive dysfunction, and increased risk of traffic and occupational accidents due to daytime sleepiness.^[7–9]^ This multidimensional health burden poses significant challenges to individual health, healthcare systems, and socioeconomic productivity.

Despite the high prevalence and well-documented consequences of SAHS, significant underdiagnosis and undertreatment persist, with many patients lacking awareness of the disorder and its unfavorable consequences. Thus, a better general knowledge of the disease and its associated risks is important. Effective patient education is crucial for promoting early recognition, facilitating diagnostic evaluation, enhancing treatment adherence, and improving self-management.

With the increasing penetration of Internet technology, the public tends to seek health information online.^[10,11]^ Short-form video platforms such as TikTok and Bilibili have become key channels for disseminating health information due to their simplicity, vivid visuals, and interactivity.^[12]^ These platforms have not only attracted millions of users seeking health advice but have also offered visually appealing, easily digestible content, making them highly attractive for medical knowledge dissemination.^[13]^ However, despite the benefits of these platforms in disseminating health information, concerns have been raised regarding the quality and reliability of their short-video content.^[14]^

However, the democratization of health content creation and dissemination carries significant risks in terms of quality control. Owing to the lack of peer review and stringent regulatory mechanisms, many videos are of varying quality and may even propagate misleading or false information.^[15]^ Video content may be produced by nonprofessionals, commercial entities promoting specific products, or healthcare practitioners with varying levels of expertise, potentially leading to oversimplification, exaggeration of treatment efficacy, promotion of unproven alternative therapies, or omission of critical risk information. While algorithmic recommendation systems enable personalized content delivery, they may create an “information cocoon” effect or prioritize engagement metrics over accuracy, thereby amplifying misleading information.^[16]^ Previous studies have documented widespread misinformation about diseases such as diabetes, cancer, and coronavirus disease 2019 on short-video platforms, with a significant proportion of these videos containing misleading or unverified content.^[17,18]^

Despite the public’s growing reliance on short videos for health guidance and the need for effective education on chronic conditions that require long-term self-management, such as sleep apnea syndrome, the reliability and educational quality of related health content on TikTok and Bilibili platforms remain unexplored. Assessing the quality of SAHS content on TikTok and Bilibili is crucial for guiding stakeholders and ensuring patients receive accurate, evidence-based guidance. To address this research gap, this study conducted a cross-sectional content analysis of SAHS-related videos on TikTok and Bilibili. Specifically, we employed validated tools such as the modified DISCERN (mDISCERN) assessment tool, Global Quality Score (GQS), and Journal of the American Medical Association benchmark criteria (JAMA) score to evaluate the quality and reliability of these short videos. Through in-depth analysis of platform content, we aim to identify gaps in existing health communication practices. Findings will provide evidence-based support for developing digital health content standards and optimizing patient education strategies.

## 2. Methods and materials

### 2.1. Ethical considerations

This study used publicly available data and did not require ethics committee approval.

### 2.2. Search strategy and data collection

**Figure 1.**
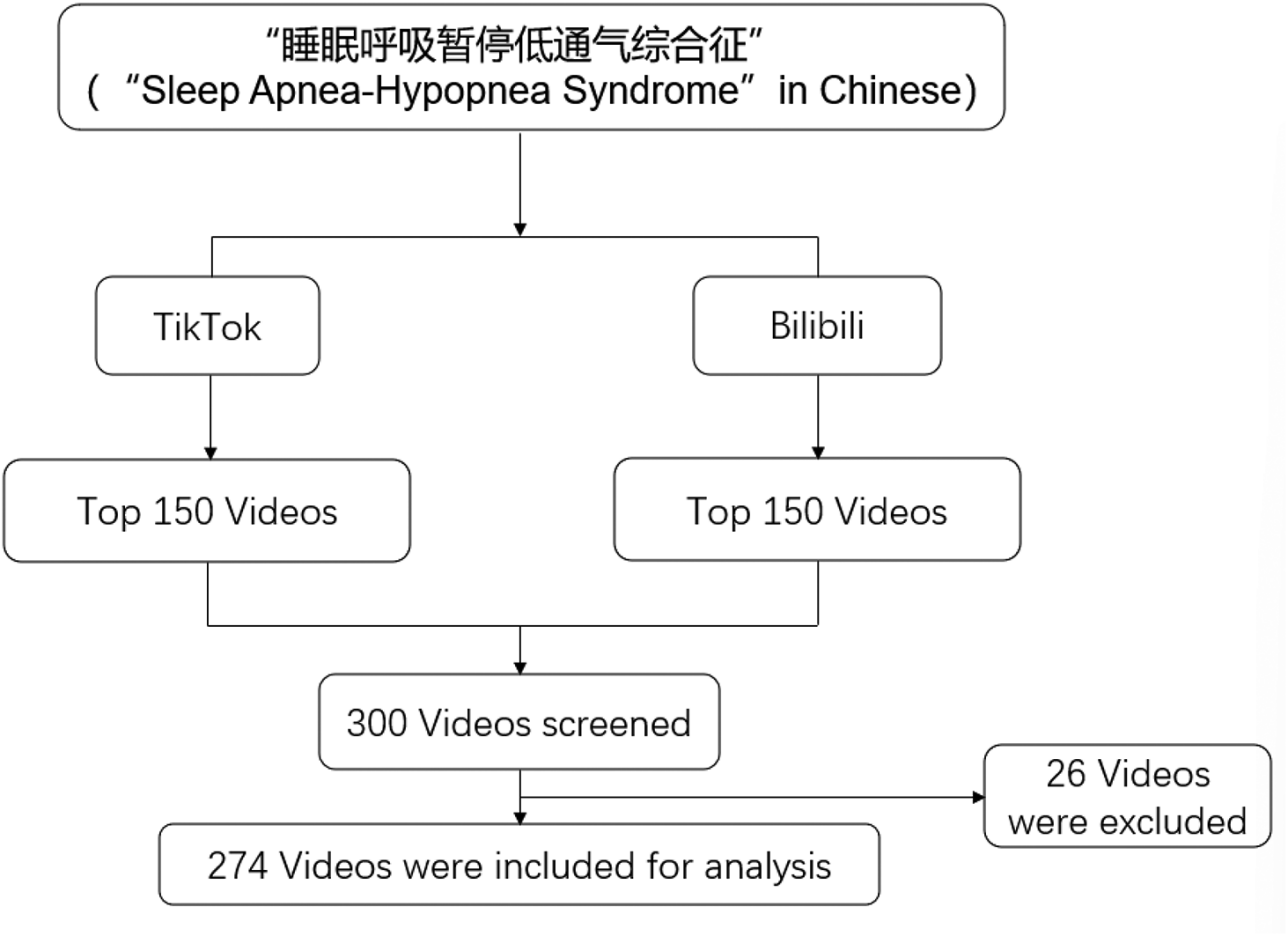
Flowchart of video selection.

This study adopted a cross-sectional, observational content analysis design and was conducted in December 2025. We used the Chinese term “睡眠呼吸暂停低通气综合征” (sleep apnea-hypopnea syndrome) as the search keyword to collect data from 2 platforms: TikTok (https://www.douyin.com) and Bilibili (https://www.bilibili.com). To minimize bias from personalized recommendation algorithms, searches were conducted as unregistered visitors without logging in, ensuring historical data or personalized algorithms did not influence results. The initial sample consisted of the top 150 videos sorted by default comprehensive ranking on each platform, which were further filtered to exclude duplicate videos, non-Chinese-language videos, and videos unrelated to the research topic (as shown in Fig. 1). We limited our analysis to the top 150 videos, a constraint supported by prior research indicating that videos ranked lower than 150 have a negligible impact on the analysis.^[19–21]^ We documented detailed information about the selected videos, including the video title, URL, uploader’s name, the content it presented, video length, number of likes, collections, and shares. All extracted data were documented in Microsoft Excel spreadsheets.

### 2.3. Uploader characteristics

The uploaders of the videos were categorized as healthcare professionals (such as physicians, nurses, and therapists), professional institutions (hospitals, medical associations, and academic organizations), or individual users (patients, caregivers, and nonmedical creators).

### 2.4. Video quality and reliability assessments

To evaluate the quality and reliability of short videos, this study employed 3 scoring tools: the GQS, the mDISCERN score, and the JAMA. These tools are widely recognized for their application in evaluating medical and health information. The GQS evaluates online video quality through 5 levels, scoring from 1 (poor) to 5 (excellent).^[22]^ This scale considered aspects such as the professionalism of the video, the comprehensiveness of the information, the clarity of the presentation, and the viewer’s ability to understand the content.^[23]^ The mDISCERN is used to evaluate the reliability of video content. Evaluators assessed each video against the following criteria: clarity, relevance, traceability, reliability, and fairness. Each of these criteria is scored on a binary scale: a “yes” response is awarded 1 point, and a “no” response is awarded 0 points. As a result, the cumulative score for a video can span from 0 to 5 points, with higher scores indicating greater reliability. This provides a quantitative metric for evaluating video reliability.^[24,25]^ The JAMA benchmark criteria, consisting of multiple dimensions such as authorship, attribution, currency, and disclosure, are employed to further appraise the quality and reliability of videos.^[26]^ Additionally, in evaluating video content, we performed binary classification (yes/no) on mentions of video content such as epidemiology, etiology, clinical manifestation, diagnosis, treatment, and prognosis. All evaluations were conducted by 2 assessors with relevant medical backgrounds, who underwent unified training prior to scoring to ensure consistency in evaluation standards and minimize bias.

### 2.5. Statistical analysis

This study employed descriptive statistics and nonparametric tests to analyze video characteristics and quality metrics. Categorical variables were presented as frequencies and percentages, whereas continuous variables were described using medians with interquartile ranges (IQRs). For intergroup comparisons, independent samples *t* tests were used for normally distributed variables and Mann–Whitney *U* tests for non-normally distributed variables. The Kruskal–Wallis *H* test was used for comparisons among 3 or more groups. When differences were statistically significant, Dunn’s post hoc test was applied for pairwise comparisons. Spearman rank correlation coefficient was used to assess the correlation between video quality scores (GQS, JAMA, and mDISCERN) and interaction metrics (likes, comments, shares, and collections). A two-tailed *P* value < .05 was considered statistically significant. All statistical analyses were performed using R software (version 4.3.2).

## 3. Results

This study aimed to analyze the characteristics, quality, and content of social media videos related to SAHS on TikTok and Bilibili. We aimed to examine how video features, uploader identities, and content categories correlate with audience interaction and video quality. Additionally, we sought to understand how these factors differ between the 2 platforms and across video sources, including healthcare professionals, professional institutions, and individual users. The following results highlight key findings in these areas, focusing on the video characteristics, content, uploader sources, and platform differences.

### 3.1. Video characteristics

We screened the top 150 videos from TikTok and Bilibili based on the inclusion and exclusion criteria, ultimately selecting a sample of 274 videos. The detailed screening process is shown in Figure 1, while Table 1 presents the characteristics of the included videos. Of the 274 videos, 150 (54.74%) videos are from the TikTok platform, and 124 (45.26%) videos are from the Bilibili platform.

**Table 1:** General characteristics, quality, and reliability of the videos.

Variables	Total (n = 274)
General information	
Video length (s), median (IQR)	96.00 (51.25–255.00)
Likes, median (IQR)	128.00 (10.00–813.50)
Collections, median (IQR)	55.00 (12.00–300.25)
Comments, median (IQR)	13.00 (1.00–117.75)
Shares, median (IQR)	73.50 (7.25–650.00)
Video content	
Epidemiology	83 (30.29%)
Etiology	145 (52.73%)
Clinical manifestation	183 (66.79%)
Diagnosis	134 (48.91%)
Treatment	183 (66.79%)
Prognosis	113 (41.24%)
Video quality	
GQS score, median (IQR)	3.00 (2.00–4.00)
JAMA, median (IQR)	2.00 (2.00–3.00)
mDISCERN score, median (IQR)	2.00 (2.00–4.00)

Data are presented as n (%) for categorical variables. Continuous variables are presented as median (IQR) because they were not normally distributed. IQR indicates the interquartile range and is shown as Q1–Q3 (25th–75th percentiles).

GQS = Global Quality Score, IQR = interquartile range, JAMA = Journal of the American Medical Association benchmark criteria, mDISCERN = modified DISCERN.

The median number of likes, collections, comments, and shares per video was 128.00 (IQR: 10.00–813.50), 55.00 (IQR: 12.00–300.25), 13.00 (IQR: 1.00–117.75), and 73.50 (IQR: 7.25–650.00). The median video length was 96.00 seconds (IQR: 51.25–255.00). In terms of video quality, the median GQS score was 3.00 (IQR: 2.00–4.00), the median mDISCERN score was 2.00 (IQR: 2.00–4.00), and the JAMA score was 2.00 (IQR: 2.00–3.00).

### 3.2. Uploader characteristics

**Figure 2.**
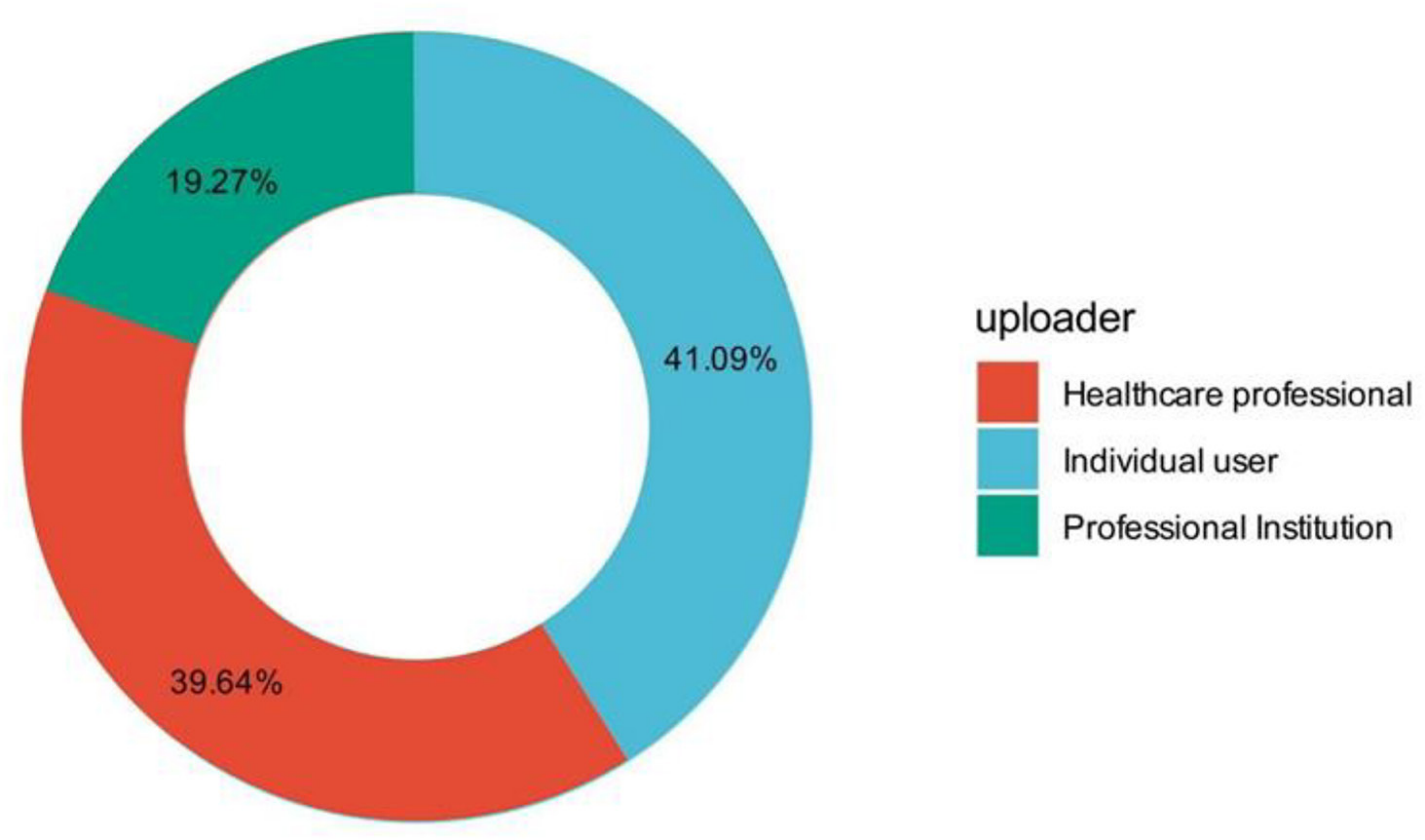
Donut chart showing the percentage of uploader types on all platforms.

**Figure 3.**
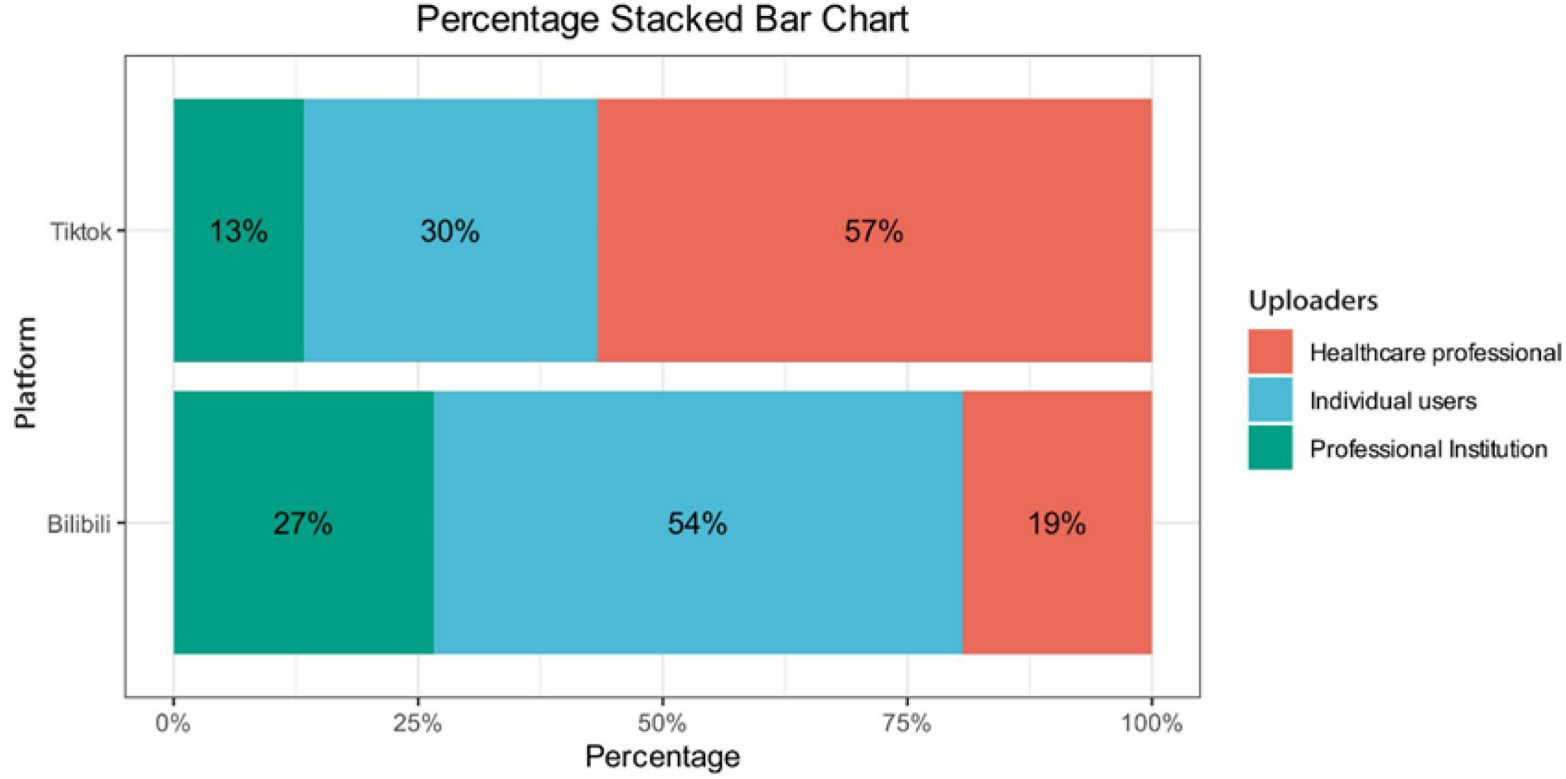
Percentage stacked bar chart showing the percentage of uploader types on different platforms (TikTok and Bilibili).

In our study, video uploaders were primarily categorized into 3 groups: healthcare professionals, professional institutions, and individual users. Among all videos, 109 (accounting for 39.64%) were uploaded by healthcare professionals, 53 (19.27%) by professional institutions, and 113 (41.09%) by individual users (Fig. 2). On Bilibili, individual users dominated with 54% of uploads, followed by professional institutions, whereas healthcare professionals had lower shares. Conversely, on TikTok, healthcare professionals were the predominant uploaders at 57%, followed by professional institutions, with individual users contributing less, as shown in Figure 3. This underscores that healthcare professionals are the primary content providers on TikTok, while individual users are the main content creators on Bilibili.

### 3.3. Video content

**Figure 4.**
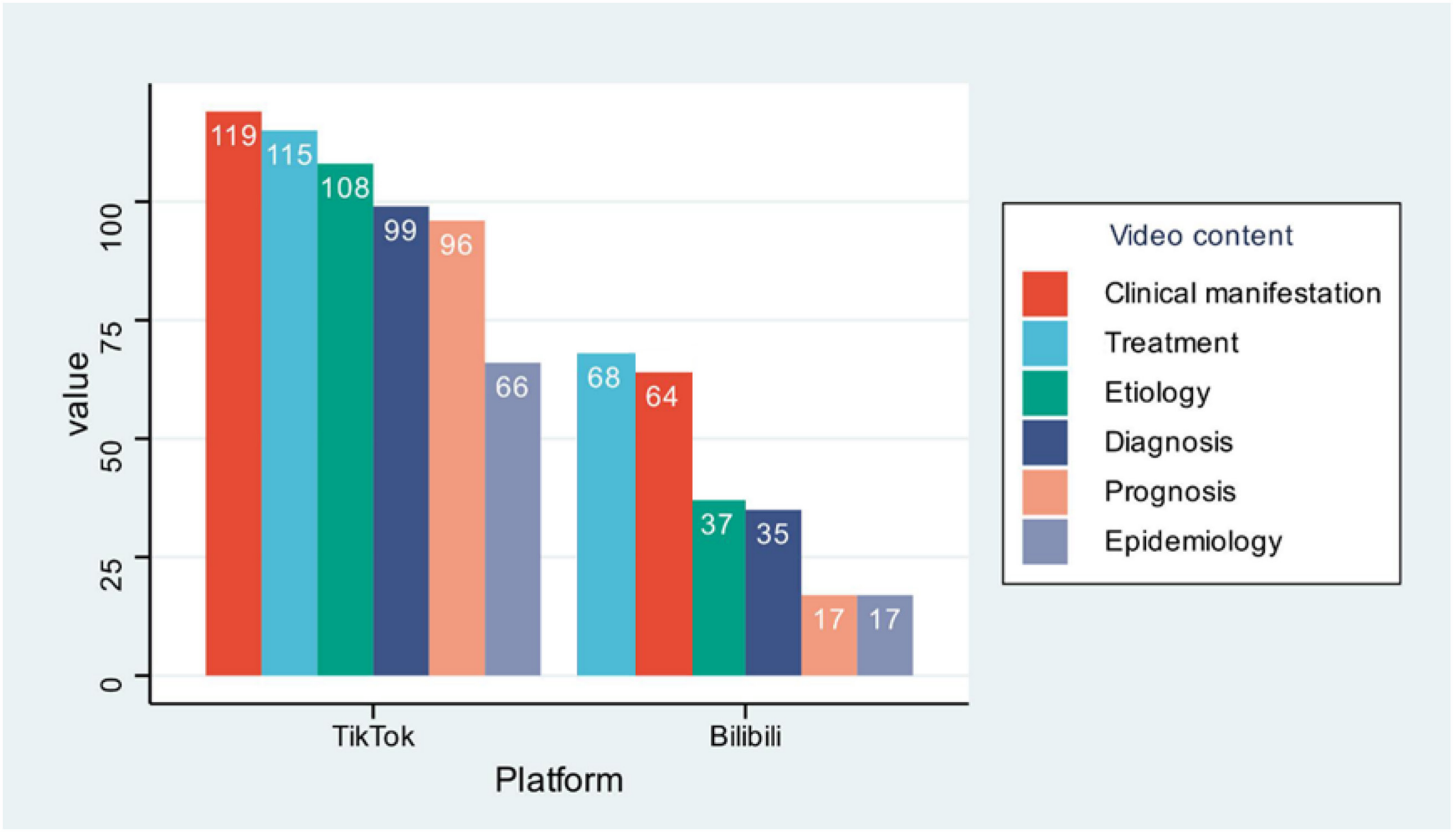
Bar chart distribution of video content on different platforms (TikTok and Bilibili), and the bars are arranged in descending order.

Among the 274 videos, 83 (30.29%) mentioned epidemiology, 145 (52.73%) mentioned etiology, 183 (66.79%) mentioned clinical manifestation, 134 (48.91%) mentioned diagnosis, 183 (66.79%) mentioned treatment, and 113 (41.24%) mentioned prognosis, as shown in Table 1 and Figure 4. This indicates that video uploaders are more inclined to disseminate information about the clinical manifestations and treatment of SAHS.

### 3.4. Comparison of features across platforms

We conducted a comparative analysis of video features across the 2 platforms, covering video length and audience interaction (likes, collections, comments, and shares). Analysis of Table 2 indicates significant differences between TikTok and Bilibili in these aspects (*P* < .001), with TikTok videos significantly shorter in length than Bilibili videos. TikTok videos exhibited superior audience interaction, receiving the highest number of likes (median: 595.00, IQR: 172.00–2249.25), collections (median: 178.50, IQR: 48.25–849.00), comments (median: 70.50, IQR: 16.00–265.00), and shares (median: 354.00, IQR: 80.00–1819.75), while Bilibili videos showed lower values across all interaction metrics. In terms of video content, videos covering clinical manifestations and treatments dominate on both platforms. Specifically, videos covering clinical manifestations account for 51.61% on Bilibili and 79.33% on TikTok, while those covering treatments represent 54.84% on Bilibili and 75.66% on TikTok. Other content categories, including etiology, diagnosis, prognosis, and epidemiology, were also covered, with consistently higher coverage on TikTok than on Bilibili (Table 2). The distribution of video content across different platforms is shown in Figure 4. Regarding video quality, the results were summarized in Table 2. The median GQS score for TikTok was 4.00 (3.00–5.00), while for Bilibili, it was 2.00 (IQR: 1.00–3.00). Regarding mDISCERN scores, TikTok had a median score of 4.00 (IQR: 2.00–5.00), whereas Bilibili had a median score of 2.00 (IQR: 1.00–2.00). As for JAMA scores, TikTok had a median score of 3.00 (IQR: 2.00–3.00), whereas Bilibili had a median score of 2.00 (IQR: 2.00–2.00). These results indicate significant differences in mDISCERN, GQS, and JAMA scores between TikTok and Bilibili, suggesting that videos on TikTok have higher quality and reliability.

**Table 2:** General information, quality, and reliability scores of SAHS videos on TikTok and Bilibili.

Variables	Bilibili (n = 124)	TikTok (n = 150)	*P*
General information			
Video length (s), median (IQR)	242.50 (75.25–688.00)	67.50 (44.00–107.25)	**<.001**
Likes, median (IQR)	8.00 (1.00–40.00)	595.00 (172.00–2249.25)	**<.001**
Collections, median (IQR)	15.00 (1.75–49.75)	178.50 (48.25–849.00)	**<.001**
Comments, median (IQR)	1.00 (0.00–5.25)	70.50 (16.00–265.00)	**<.001**
Shares, median (IQR)	7.00 (0.75–44.25)	354.00 (80.00–1819.75)	**<.001**
Video content			
Epidemiology	17 (13.71%)	66 (44%)	–
Etiology	37 (29.84%)	108 (72%)	–
Clinical manifestation	64 (51.61%)	119 (79.33%)	–
Diagnosis	35 (28.23%)	99 (66%)	–
Treatment	68 (54.84%)	115 (75.66%)	–
Prognosis	17 (13.71%)	96 (64%)	–
Video quality			
GQS score, median (IQR)	2.00 (1.00–3.00)	4.00 (3.00–5.00)	**<.001**
JAMA, median (IQR)	2.00 (2.00–2.00)	3.00 (2.00–3.00)	**<.001**
mDISCERN score, median (IQR)	2.00 (1.00–2.00)	4.00 (2.00–5.00)	**<.001**

Categorical variables are presented as n (%). Continuous variables are presented as median (IQR) because they were not normally distributed. IQR indicates the interquartile range and is shown as Q1–Q3 (25th–75th percentiles). *P* values for continuous variables were calculated using the Mann–Whitney *U* test (non-normally distributed data).

GQS = Global Quality Score, IQR = interquartile range, JAMA = Journal of the American Medical Association benchmark criteria, mDISCERN = modified DISCERN, SAHS = sleep apnea-hypopnea syndrome.

### 3.5. Comparison of features across different video sources

We further compared the features and quality of videos based on uploader identities. Table 3 presents a detailed analysis of video characteristics and quality, all of which were significantly different. Videos uploaded by healthcare professionals were notably shorter in video length compared with those of other identities. In terms of video popularity, healthcare professional-uploaded videos demonstrated excellent metrics with higher interaction levels regarding likes, collections, comments, and shares. A comparison of video quality and reliability among uploaders with different identity types revealed significant variations in GQS, mDISCERN, and JAMA scores, as presented in Table 3. Healthcare professional-uploaded videos performed notably better in quality and reliability, with GQS, mDISCERN, and JAMA scores of 4 (IQR: 4–4), 4 (IQR: 2–5), and 3 (IQR: 2–3), respectively. They had significantly higher mDISCERN, GQS, and JAMA scores compared with videos uploaded by professional institutions and individual users, with no significant differences between the latter two groups.

**Table 3:** Characteristics, quality, and reliability of SAHS videos by different uploaders on TikTok and Bilibili.

Variables	Healthcare professionals (n = 109)	Professional institutions (n = 53)	Individual users (n = 112)	*P*
Video length (s), median (IQR)	71.00 (44.00–108.00)	153.00 (61.00–291.00)	151.00 (62.25–584.25)	**<.001**
Likes, median (IQR)	286.00 (86.00–1147.00)	46.00 (9.00–447.00)	41.50 (3.00–554.25)	<.001
Collections, median (IQR)	115.00 (21.00–598.00)	36.00 (17.00–194.00)	37.00 (3.75–234.25)	.002
Comments, median (IQR)	51.00 (7.00–172.00)	4.00 (0.00–51.00)	7.00 (0.00–91.25)	**<.001**
Shares, median (IQR)	151.00 (17.00–1136.00)	49.00 (9.00–199.00)	29.50 (2.00–345.25)	.001
GQS score, median (IQR)	4.00 (4.00–4.00)	3.00 (2.00–5.00)	3.00 (2.00–3.00)	**<.001**
JAMA score, median (IQR)	3.00 (2.00–3.00)	2.00 (2.00–4.00)	2.00 (1.00–2.00)	**<.001**
mDISCERN score, median (IQR)	4.00 (2.00–5.00)	3.00 (2.00–5.00)	2.00 (1.00–2.00)	**<.001**

Continuous variables are presented as median (IQR). *P* values were calculated using the Kruskal–Wallis test for comparisons across the 3 uploader groups.

GQS = Global Quality Score, IQR = interquartile range, JAMA = Journal of the American Medical Association benchmark criteria, mDISCERN = modified DISCERN, SAHS = sleep apnea-hypopnea syndrome.

### 3.6. Correlation analysis between video features and quality

**Figure 5.**
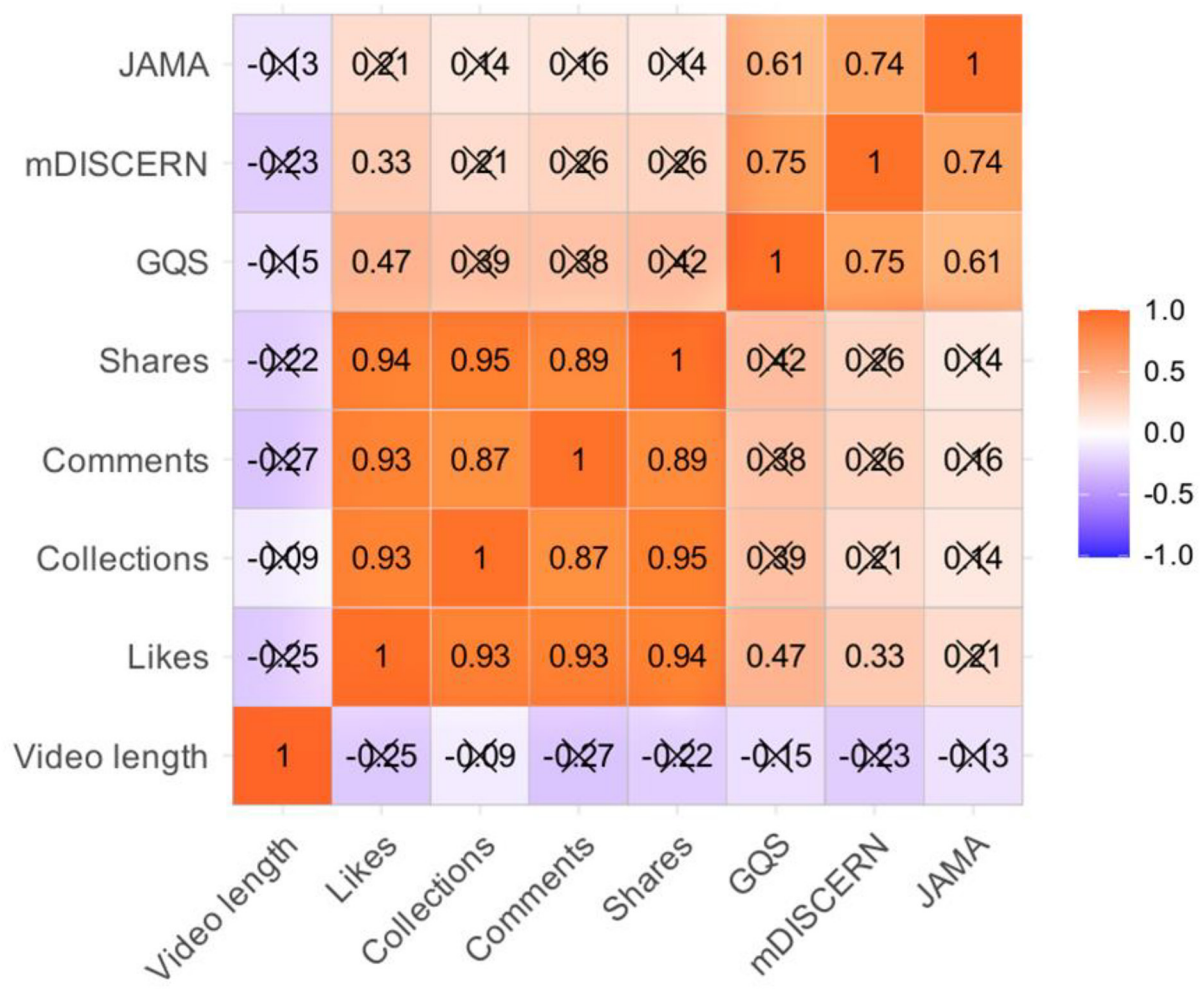
Spearman correlation analysis among different video variables, GQS, JAMA, and mDISCERN score concerning SAHS videos. GQS = Global Quality Score, JAMA = Journal of the American Medical Association benchmark criteria, SAHS = sleep apnea-hypopnea syndrome.

Spearman correlation analysis was used to explore the correlation among different video interaction metrics (such as likes, comments, collections, and shares) and GQS scores, mDISCERN scores, and JAMA scores in SAHS-related videos. The results, depicted in Figure 5, revealed strong positive correlations among likes, comments, shares, and collections. For instance, the correlation coefficients between likes and comments, likes and shares, and likes and collections were 0.93, 0.94, and 0.93, respectively. In contrast, video length (seconds) had no correlation with other variables, except for the correlation coefficients between likes and GQS score and likes and mDISCERN score, which were 0.47 and 0.33, respectively. No correlations were observed between the other interaction indicators and the GQS score, mDISCERN score, or JAMA score.

In summary, the study found that TikTok videos were shorter and had significantly higher audience engagement (likes, shares, comments, and collections) compared with Bilibili videos. Healthcare professionals were the most prevalent uploaders on TikTok, whereas individual users dominated Bilibili. In terms of content, clinical manifestations and treatment information were most commonly featured on both platforms, with TikTok videos showing higher coverage of topics like etiology, diagnosis, prognosis, and epidemiology. When it came to video quality, TikTok videos scored higher on all quality metrics, including GQS, mDISCERN, and JAMA scores, indicating better reliability and content quality. Furthermore, healthcare professionals’ videos consistently outperformed those uploaded by professional institutions and individual users in terms of both content quality and audience interaction. This analysis emphasizes the influence of uploader identity and platform differences on video features, interaction metrics, and overall quality.

## 4. Discussion

In recent years, the rapid growth of short-video platforms has made social media an important source of health information for the public. TikTok and Bilibili, which are currently the dominant short-video platforms in China, play an increasingly important role in disseminating health information.^[27,28]^ This aligns with the broader trend of social media playing an increasingly significant role in healthcare, with approximately 80% of internet users accessing health information online.^[29,30]^ Therefore, evaluating the quality and reliability of health information on these platforms is crucial. Our study conducted a comprehensive and systematic evaluation of content related to SAHS on TikTok and Bilibili, the 2 major short-video platforms currently dominant in China. We analyzed the quality and reliability of 274 video messages utilizing 3 validated assessment tools: the JAMA benchmark criteria, the GQS, and the mDISCERN instrument. Our findings revealed significant differences between the 2 platforms in terms of video characteristics, content coverage, uploader attributes, and audience engagement metrics. Overall, videos on the TikTok platform outperform those on Bilibili across all metrics, and the professional background of video uploaders plays a decisive role in determining content quality.^[31]^ Furthermore, by integrating the GQS, JAMA, and mDISCERN tools, this study provides a more systematic and objective assessment of health information videos, offering valuable insights for public health policy development, content regulation on short-video platforms, and health education initiatives.^[32,33]^ The findings provide insights into the current landscape of video content related to sleep apnea hypoventilation syndrome, revealing that social media platforms – particularly TikTok – offer patients a convenient avenue to access medical knowledge and seek social support.^[34]^ These findings also provide empirical support for optimizing health information dissemination strategies.

Our analysis confirms that TikTok and Bilibili play distinct roles in disseminating SAHS-related content, reflecting their unique platform ecosystems. TikTok videos are considerably shorter than those on Bilibili, yet exhibit higher user interaction across all metrics (likes, saves, comments, and shares), attributable to platform-specific characteristics. TikTok’s short videos typically range from 15 to 60 seconds, facilitating rapid dissemination. In contrast, Bilibili’s longer videos demand greater user patience, potentially reducing interaction. This suggests that its user base may place greater emphasis on comprehensive knowledge rather than concise formats, though this does not necessarily translate into broader dissemination or interaction. This finding aligns with trends observed in previous scholarly research on short-form videos.^[35]^

Notably, healthcare professionals were the primary uploaders on TikTok (57%), whereas individual users dominated on Bilibili (54%). This disparity may result from TikTok’s stringent professional certification and rigorous video review processes, whereas Bilibili imposes fewer requirements for professional certification. Conversely, while Bilibili’s openness to user-generated content fosters diverse perspectives, it may compromise average information quality, resulting in significantly lower GQS, mDISCERN, and JAMA scores compared with TikTok.

The majority of video content in the study focused on clinical manifestations (66.79%) and treatment (66.79%), followed by etiology (52.73%) and diagnosis (48.91%). This reflects public attention to symptom recognition and treatment options while revealing significant coverage gaps in epidemiology (30.29%), prognosis (41.24%), and preventive measures. Such omissions may limit viewers’ holistic understanding of SAHS as a chronic systemic disease requiring lifelong management. Furthermore, videos exhibit a pronounced tendency to emphasize treatment modalities over risk factor interventions or phased management approaches, mirroring patterns observed in prior video analyses of other conditions.^[35,36]^ This tendency may inadvertently foster a passive rather than preventive health mindset among viewers, particularly when content lacks nuanced discussion of individualized treatment applicability. Therefore, healthcare professionals should be encouraged to produce higher-quality SAHS public education videos focused on modifiable risk factors to align with preventive care objectives and reduce future clinical burdens. Such initiatives not only enhance information quality but also empower patients for early intervention, ultimately alleviating the burden of SAHS cases. Fundamentally, as these short videos increasingly shape patient expectations and behaviors, clinicians must recognize their influence, proactively counter misinformation, and leverage high-quality content to enhance patient education and adherence to evidence-based treatments.

In terms of uploaders, we found that videos produced by healthcare professionals consistently outperformed content uploaded by professional organizations and general users across all quality metrics, such as GQS, JAMA, and mDISCERN scores. This further underscores the critical role of medical expertise in ensuring the scientific accuracy, reliability, and educational value of health communication. Videos uploaded by healthcare professionals demonstrate exceptional performance on TikTok, not only achieving the highest engagement rates but also indicating that the platform’s mechanisms for promoting verified content can simultaneously enhance dissemination reach and information credibility. Notably, TikTok’s strong performance in the author identity dimension of the JAMA score further validates its rigorous review mechanisms. Therefore, it is recommended that physicians on all platforms incorporate guideline references and the latest research findings into their videos to enhance professionalism and credibility.

However, the lack of significant quality differences between institutional and individual uploads raises concerns about consistency in content review across organizational channels. This also highlights the potential risk that nonprofessional uploaders, while capable of creating highly engaging content, may lack scientific rigor. This result aligns with previous studies on other disease-related videos.^[19,21]^ Researchers attributed these circumstances to TikTok’s recommendation mechanism, which dictates that videos with more likes are more likely to be recommended.^[21]^ Consequently, low-quality popular videos may gain disproportionate popularity, further widening the gap between video quality and popularity.

Correlation analysis revealed a significant positive correlation among likes, comments, shares, and saves, indicating that videos with more likes also tend to be shared and saved frequently. This aligns with prior research findings that user engagement metrics are interdependent on short-video platforms.^[37]^ However, these metrics showed only weak to moderate correlations with GQS and mDISCERN scores, and no association with JAMA scores. Videos with high interactivity did not necessarily possess high scientific quality, a finding consistent with prior studies on videos related to other diseases.^[35,37]^ This disconnect highlights the impact of platform algorithms prioritizing entertainment over educational content in recommendation mechanisms. Without deliberate weighting of quality metrics in recommendation systems, scientifically rigorous videos may struggle to gain visibility, thereby perpetuating the spread of attention-grabbing yet misleading information. Consequently, platforms should establish video filtering mechanisms that prioritize the display of professional and high-quality videos in search results, thereby ensuring the spread of precise knowledge.

This study employed a dual-platform comparative design, multidimensional quality assessment, and systematic uploader influence analysis, offering significant advantages. Furthermore, it represents China’s first research analyzing short-video quality across 2 social media platforms within the SAHS domain. However, there are some limitations in our study. First, cross-sectional data only captures conditions at a specific point in time, potentially failing to reflect content trends or dynamic changes in platform policies. Second, while this study quantitatively analyzed the volume of video comments, it did not conduct an in-depth qualitative analysis of comment content. Future research should delve into the underlying meaning of audience feedback, such as whether it reflects misinformation, user engagement, or skepticism, as these factors could offer deeper insights into how viewers perceive and interact with SAHS-related content. Incorporating patient perspectives, for example, by exploring how videos created by SAHS patients influence viewers’ understanding and health decision-making, may provide a more comprehensive dimension for evaluating video quality. Third, this analysis is confined to Chinese-language platforms, limiting the applicability of its conclusions to other languages and cultures. Future research should include cross-platform comparisons with global platforms like YouTube. Finally, video content quality scoring relies on manual evaluation, introducing potential subjective bias. Integrating AI-driven analytical tools could offer more objective and efficient solutions for video content analysis, representing a promising direction for future research.

## 5. Conclusion

This study assessed the quality of 274 videos related to SAHS on TikTok and Bilibili using the GQS, mDISCERN, and JAMA scoring systems. Videos on TikTok were slightly better than those on Bilibili, but the overall video quality of SAHS content on both platforms still needs improvement. Videos uploaded by healthcare professionals demonstrated higher quality and reliability, offering viewers more valuable information. Given the rise of short videos, healthcare professionals and healthcare institutions must ensure the provision of high-quality content related to SAHS. Additionally, short-form video platforms should strengthen their monitoring and review mechanisms. When seeking medical information, patients should exercise caution when viewing videos on Bilibili and TikTok.

## Author contributions

**Conceptualization:** Shan Wang, Ying Mao, Fang Wang, Jiaqi Li, Zhenxing Zhang.

**Data curation:** Shan Wang, Ying Mao, Fang Wang, Jiaqi Li, Zhenxing Zhang.

**Formal analysis:** Shan Wang, Ying Mao, Fang Wang, Jiaqi Li, Zhenxing Zhang.

**Funding acquisition:** Shan Wang.

**Investigation:** Shan Wang, Ying Mao, Fang Wang, Jiaqi Li, Zhenxing Zhang.

**Methodology:** Shan Wang, Ying Mao, Fang Wang, Jiaqi Li, Zhenxing Zhang.

**Project administration:** Shan Wang, Zhenxing Zhang.

**Resources:** Shan Wang, Ying Mao, Fang Wang, Zhenxing Zhang.

**Software:** Shan Wang, Jiaqi Li, Zhenxing Zhang.

**Supervision:** Shan Wang.

**Validation:** Shan Wang, Ying Mao, Fang Wang, Jiaqi Li, Zhenxing Zhang.

**Visualization:** Shan Wang, Ying Mao, Fang Wang, Jiaqi Li, Zhenxing Zhang.

**Writing – original draft:** Shan Wang, Zhenxing Zhang.

**Writing – review & editing:** Shan Wang, Zhenxing Zhang.
